# Interdental Plaque Microbial Community Changes under In Vitro Violet LED Irradiation

**DOI:** 10.3390/antibiotics10111348

**Published:** 2021-11-04

**Authors:** Dan Wang, Takayuki Nambu, Hiroaki Tanimoto, Naohiro Iwata, Kazushi Yoshikawa, Toshinori Okinaga, Kazuyo Yamamoto

**Affiliations:** 1 Department of Operative Dentistry, Graduate School of Dentistry, Osaka Dental University, 8-1, Kuzuha-Hanazono, Hirakata, Osaka 573-1121, Japan; wang-d@cc.osaka-dent.ac.jp; 2 Department of Bacteriology, Osaka Dental University, 8-1, Kuzuha-Hanazono, Hirakata, Osaka 573-1121, Japan; 3 Department of Operative Dentistry, Osaka Dental University, 8-1, Kuzuha-Hanazono, Hirakata, Osaka 573-1121, Japan; tanimoto@cc.osaka-dent.ac.jp (H.T.); iwata@cc.osaka-dent.ac.jp (N.I.); kazushi@cc.osaka-dent.ac.jp (K.Y.); yamamoto@cc.osaka-dent.ac.jp (K.Y.)

**Keywords:** oral microbiota, violet light, 16S rRNA gene, high-throughput sequencing

## Abstract

Oral microbiome dysbiosis has important links to human health and disease. Although photodynamic therapy influences microbiome diversity, the specific effect of violet light irradiation remains largely unknown. In this study, we analyzed the effect of violet light-emitting diode (LED) irradiation on interdental plaque microbiota. Interdental plaque was collected from 12 human subjects, exposed to violet LED irradiation, and cultured in a specialized growth medium. Next-generation sequencing of the 16S ribosomal RNA genes revealed that α-diversity decreased, whereas β-diversity exhibited a continuous change with violet LED irradiation doses. In addition, we identified several operational taxonomic units that exhibited significant shifts during violet LED irradiation. Specifically, violet LED irradiation led to a significant reduction in the relative abundance of *Fusobacterium* species, but a significant increase in several species of oral bacteria, such as *Veillonella* and *Campylobacter*. Our study provides an overview of oral plaque microbiota changes under violet LED irradiation, and highlights the potential of this method for adjusting the balance of the oral microbiome without inducing antibiotic resistance.

## 1. Introduction

Human oral microbiota are estimated to comprise more than 700 diverse phylotypes, of which more than 50% have not been cultivated; however, only approximately 100 phylotypes are found in a typical individual [1,2]. Housing the second-most diverse microbial community in the body [3], the oral habitat is the most stable microbiome, with a higher alpha diversity than other parts of the body [3,4]. Under normal circumstances, the proliferation of pathogenic oral microorganisms would be suppressed when oral microbiota form a symbiotic biofilm, making a substantial contribution to a healthy oral state [5,6]. However, factors such as sugar consumption, antibiotic treatment, and excessive exposure to carbohydrates can induce dysbiosis of the oral microbiota, leading to dental caries, gingivitis, and periodontitis [7,8,9]. Furthermore, oral infections, especially periodontitis, have been shown to influence the process and pathogenesis of several systemic diseases, such as bacterial pneumonia, cardiovascular disease, low birth weight, and diabetes mellitus, suggesting that the oral microbiome may potentially serve as an essential part in the etiology of some systemic diseases [10,11,12,13]. In elderly people, the type of tongue microbiota is significantly associated with an increased mortality risk from aspiration pneumonia [14,15]. Moreover, the accumulation of dental plaque, which is an oral biofilm attached to the tooth surface with polymer matrixes [16], is generally thought to elevate the risk of periodontal diseases, especially gingivitis, by shifting microbiota to predominantly anaerobic species that can cause inflammation [17,18]. Conversely, some systemic diseases, such as systemic lupus erythematosus and rheumatoid arthritis, may result in the alterations in the composition of human microbiota [19,20]. Although the recent development and subsequent advancements in next-generation sequencing technologies have contributed to rapid progress in the analysis of bacterial diversity patterns [21], potential approaches for transforming oral microbiota into a healthy state have remained largely unexamined because of the various environmental factors that impact microbiota, as well as microbial community complexity.

In previous studies, we reported that compounds such as nitric oxide modulate cultured dental plaque microbiota in vitro [22], and that tongue brushing can alter the microbiome of the tongue coating and saliva in healthy individuals [23]. In bacterial communities outside of the oral cavity, 5-aminolevulinic acid-mediated photodynamic therapy (PDT) resulted in an increase in microbiome diversity in the pilosebaceous units of severe acne [24]. In fact, medical lasers are currently being investigated to treat infectious oral diseases [25,26,27]. Much scientific evidence has also confirmed the bactericidal efficacy of several individual types of visible light on periodontal pathogens, even without the presence of exogenous photoactivated compounds [28,29,30,31,32]. Moreover, blue light (400–500 nm) affects the bacterial composition and viability of microbial biofilms in vitro, including *Porphyromonas gingivalis, Prevotella* species, *Fusobacterium nucleatum* subspecies, *Fusobacterium periodonticum, Streptococcus sanguinis*, and *Actinomyces naeslundii* [28,29]. More specifically, relatively short wavelengths of light close to 405 nm, which are typically defined as the violet range of visible light, have exhibited a bactericidal effect on selected medically important Gram-positive and Gram-negative bacteria [30,31,32]. However, the mechanism by which violet light irradiation induces shifts in bacterial microbiota remains largely unknown.

Therefore, the focus of this in vitro study was violet-light PDT generated from a 400–410 nm light-emitting diode (LED) light source that does not require the addition of exogenous photosensitizer chemicals. The aim of this study was to explore the effect of violet LED irradiation over the entire composition of the oral bacterial community in interdental plaque and obtain empirical data to support community structure control via violet LED irradiation.

## 2. Results

### 2.1. Violet LED Irradiation Partly Suppresses the Growth of Plaque Microorganisms 

**Figure 1:**
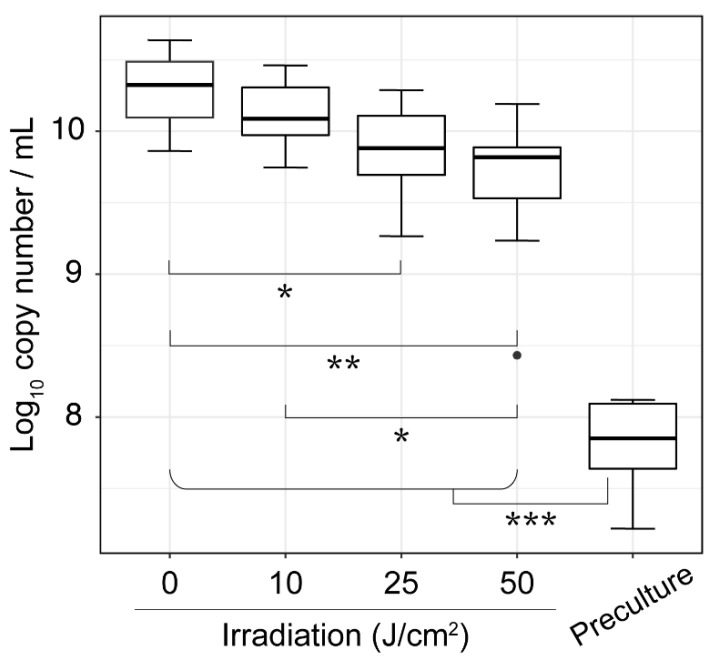
Changes in total bacterial density with increasing violet LED irradiation dose. The total number of bacterial DNA was estimated using universal bacterial 16S rRNA primers for PCR quantification. Statistical significance is indicated by horizontal lines (* *p* < 0.05, ** *p* < 0.001, *** *p* < 0.0001, Wilcoxon rank-sum test).

We assessed the effects of violet LED irradiation on dental plaque microorganisms. Dental plaque samples collected from 12 individuals were irradiated with a violet LED at irradiation doses of 0, 10, 25, or 50 J/cm^2^, and then cultured anaerobically in SHI medium for 20 h. After DNA extraction from the pre- and post-irradiated samples, the concentration of total bacterial DNA was assessed by 16S rDNA real-time polymerase chain reaction (PCR) quantification. As shown in Figure 1, the samples irradiated with doses greater than or equal to 25 J/cm^2^ reported significantly lower total bacterial densities than nonirradiated samples based on the Wilcoxon rank-sum test (*p* < 0.05).

### 2.2. In Vitro Violet LED Irradiation Shifts the Dental Plaque Microbial Community Composition 

**Figure 2:**
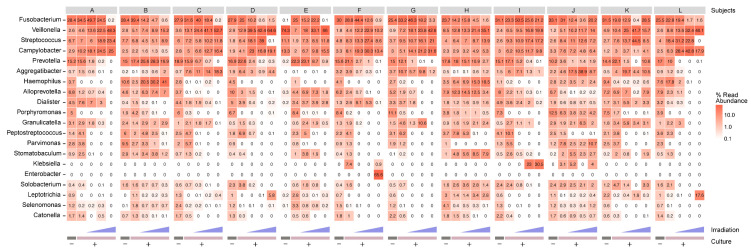
Heatmap illustrating the 20 most abundant bacterial genera after violet LED irradiation and subsequent in vitro cultivation. Values shown in each cell indicate the relative abundance as a percentage, according to sequencing of the V3–V4 region of 16S rRNA gene amplicons. Colors represent the frequency on a log10 scale. The columns are shown in order from left to right for each subject: preculture, 0, 10, 25, 50 J/cm^2^ after incubation.

The V3-V4 region of 16S rRNA gene amplicons from the extracted DNA samples underwent high-throughput sequencing and were processed using QIIME 2. As a result of paired-end assembly, quality filtering, and removal of chimera, 8,045,286 sequences were clustered in 3685 of the identified amplicon sequence variants (ASVs). Sequence counts in plaque samples ranged from 41,532 to 340,416, with an average of 134,088.1 (±39,973.7 SD) per sample. Rarefaction curves corresponding to species richness approached saturation plateaus at a sequencing depth of approximately 30,000, indicating that all samples contained reads of sufficient quality (Supplementary Material Figure S1). The structural diversity of the microbial community was analyzed using amplicon sequencing; the 20 most abundant genera and the relative population of order are shown in Figure 2 and Figure S2. The microbiota of each subject showed complex changes with LED irradiation, with *Fusobacterium*, *Prevotella*, and *Alloprevotella* abundance exhibiting an apparent reduction with increasing LED intensity in the majority of samples tested. In contrast, the relative proportions of *Veillonella* and *Campylobacter* increased with LED irradiation.

**Figure 3:**
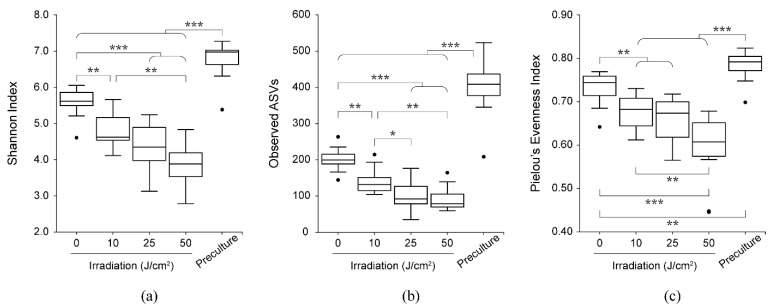
Comparisons of alpha diversity of precultured and irradiated samples at each dose. Boxplots of alpha diversity indices are displayed by (**a**) Shannon index, (**b**) observed ASVs, and (**c**) Pielou’s evenness index (* *p* < 0.05, ** *p* < 0.01, *** *p* < 0.001, Kruskal–Wallis test).

**Figure 4:**
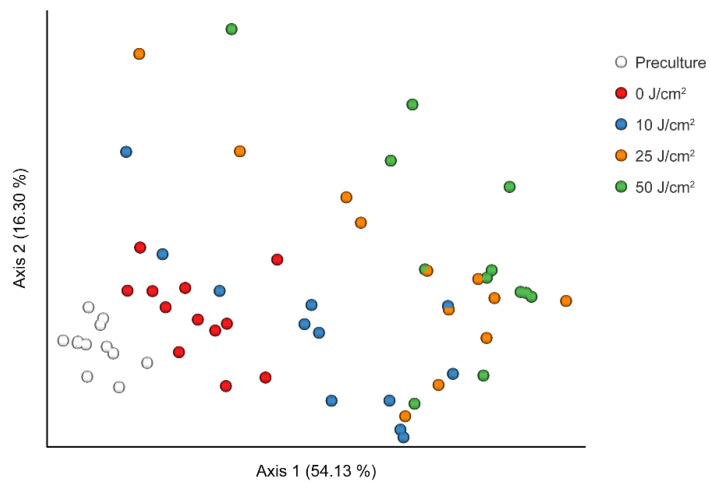
Principal coordinate analysis of weighted Unifrac distances between samples (*p* = 0.001, pairwise permutational analysis of variance).

**Figure 5:**
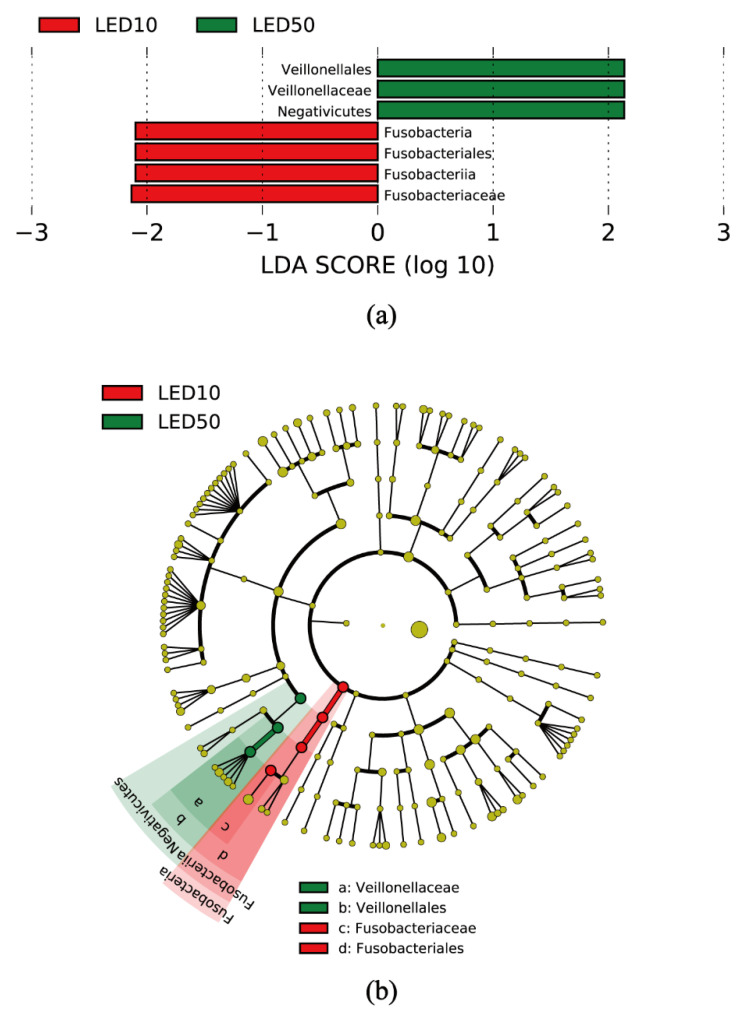
Linear discriminant analysis effect size identification of the most differentially abundant taxa between plaque microbiotas with 10 J/cm^2^ and 50 J/cm^2^ of violet LED irradiation. (**a**) Histogram of linear discriminant analysis scores (log10) computed for features with differential abundance in samples exposed to 10 J/cm^2^ and 50 J/cm^2^ of violet LED irradiation (red, 10 J/cm^2^; green, 50 J/cm^2^). (**b**) Taxonomic representation of statistically and biologically consistent differences in samples exposed to 10 J/cm^2^ and 50 J/cm^2^ of violet LED irradiation. Each filled circle represents a phylotype. The name of phylum and class are indicated on the cladogram, with the order or family given in the panel below. The diameter of each circle is proportional to the taxon abundance.

According to the Shannon index, observed ASVs, and Pielou’s evenness index, the alpha (intrasample) diversity of the community decreased significantly with violet LED irradiation dose (Figure 3a–c). A further comparison of samples cultured on SHI medium with and without irradiation revealed significant differences in the Shannon index, observed ASVs, and Pielou’s evenness index. Beta (intercommunity) diversity was altered in accordance with the violet LED irradiation dose (Figure 4 and Figure S3). The five communities showed a relatively significant separation in the plot based on principal coordinate analysis. We then performed linear discriminant analysis effect size to assess the differences in bacterial distribution. At the taxonomic level of family, a significant decrease in the relative ratio of *Fusobacteriaceae* was observed with violet LED irradiation of 10 J/cm^2^, whereas an increase in the relative ratio of *Veillonellaceae* was found with irradiation of 50 J/cm^2^ (Figure 5).

### 2.3. Violet LED Irradiation Shifts the Relative Abundance of Specific Oral Bacteria

**Figure 6:**
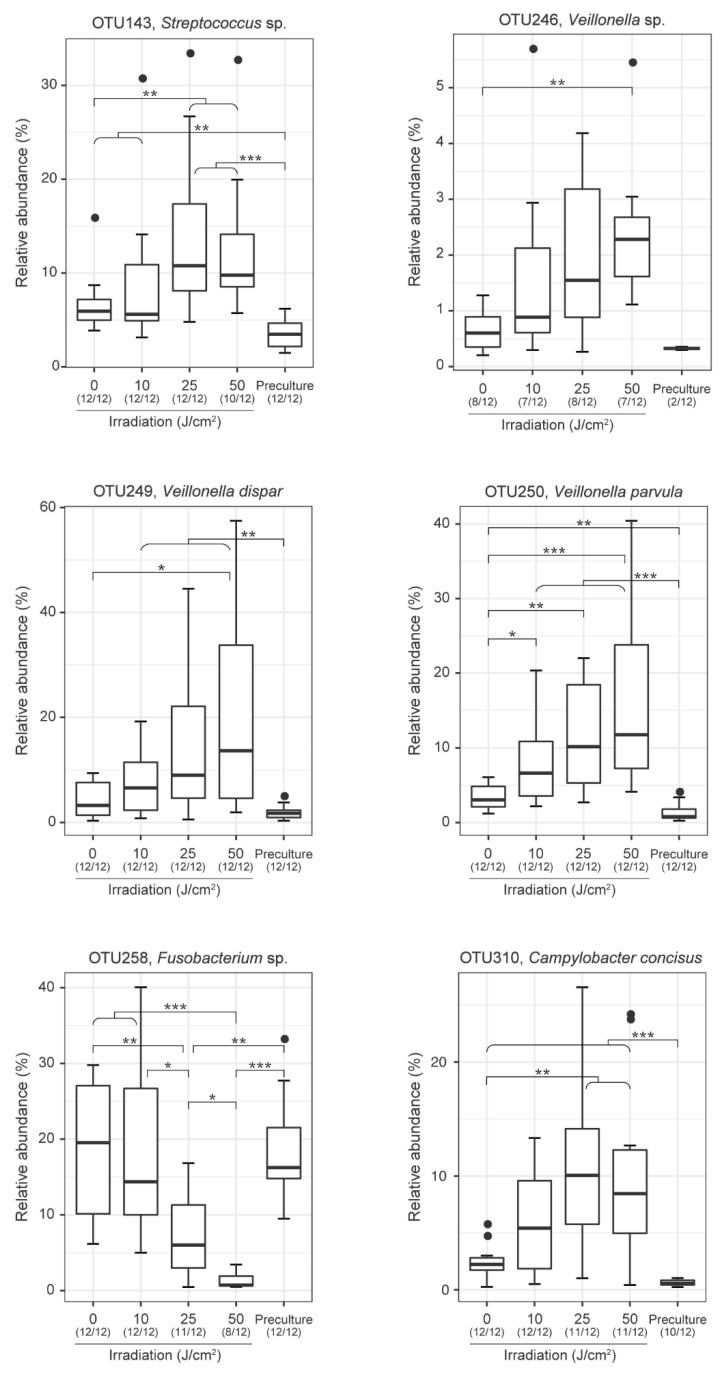
Relative abundance of the six representative operational taxonomic units (OTUs) altered by violet LED irradiation (* *p* < 0.05, ** *p* < 0.01, *** *p* < 0.001, Wilcoxon rank-sum test).

**Figure 7:**
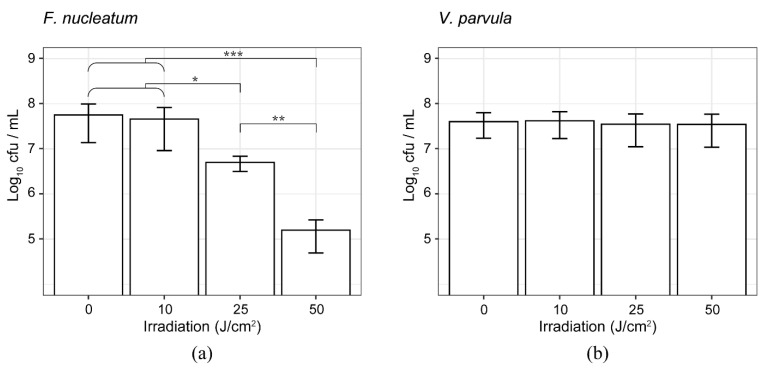
Effect of violet LED irradiation on the bacterial cell viability. (**a**) Bacterial viability of *F. nucleatum* subsp. *polymorphum* at different violet LED irradiation doses. Each value is calculated from quadruplicate assays, presented as the mean ± standard deviation. (**b**) Bacterial viability of *V. parvula* at different violet LED irradiation doses. Each value is calculated from quintuplicate assays, presented as the mean ± standard deviation (* *p* < 0.05, ** *p* < 0.01, *** *p* < 0.001, Student’s *t*-test).

The present results suggest that interdental plaque is a composition of oral bacteria with violet LED sensitivities. As shown in Figure 6, six operational taxonomic units (OTUs) with statistically significant differences were identified using the Wilcoxon rank-sum test through the analysis of the samples collected from 12 subjects. The relative proportions of *Fusobacterium* sp. decreased, whereas the relative abundance of *Streptococcus* sp., *Veillonella* sp., *Veillonella dispar, Veillonella parvula,* and *Campylobacter concisus* increased with the irradiation dose of violet LED light. As for the genus *Fusobacterium*, *F. nucleatum* subsp. *animalis*, *F. nucleatum* subsp. *vincentii*, and *F. periodonticum* were significantly decreased according to Fisher’s exact test (Table S1). In fact, as shown in Figure 7a, when irradiated with violet LED light, the cultured *F. nucleatum* subsp. *polymorphum* exhibited a significantly decreased colony forming unit (CFU) count. In contrast, cultured *V. parvula* reported no significant decrease in the CFU count when irradiated with violet LED light (Figure 7b). Thus, the increased relative proportions in the community of some bacterial species, such as *V. parvula*, may indicate a mechanism against a bactericidal activity of violet LED irradiation. In addition to the species listed above, *Prevotella* sp., *Solobacterium moorei*, and *F. periodonticum* were identified as altered OTUs according to Fisher’s exact test.

### 2.4. Oxidative Stress Was Induced by Violet LED Irradiation in F. nucleatum

**Figure 8:**
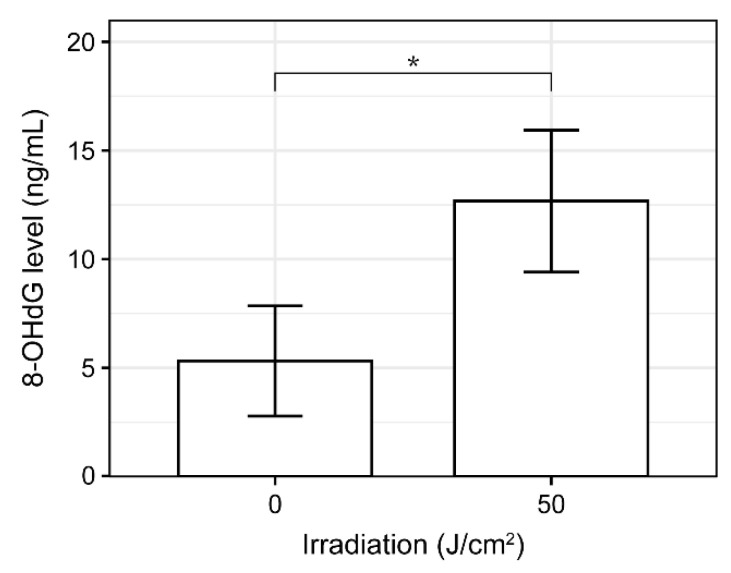
Oxidative stress induced by violet LED irradiation of *F. nucleatum* subsp. *polymorphum* (*n* = 3) (* *p* < 0.05, Student’s *t*-test).

Here, we investigated whether DNA oxidation occurred in *F. nucleatum* subsp. *polymorphum* upon violet LED irradiation. DNA was extracted from *F. nucleatum* irradiated with violet LED light, and the accumulation of 8-hydroxy-2-deoxyguanosine (8-OHdG) damage in DNA was analyzed by enzyme-linked immunosorbent assay (ELISA). The DNA of *F. nucleatum* subsp. *polymorphum* irradiated with 50 J/cm^2^ of violet LED light manifested a significantly higher ratio of 8-OHdG than the nonirradiated group, indicating that DNA was oxidatively damaged by LED irradiation (Figure 8).

## 3. Discussion 

In this study, we developed a simple in vitro system for exploring the effect of violet LED irradiation on dental plaque microbiota in individuals. Previous studies have shown that violet LED irradiation can inactivate a range of medically important bacteria [30,31,32]. Our results showed that violet LED irradiation of plaque-derived samples incubated in SHI medium led to a reduction in total bacterial density, indicating that irradiation suppressed the growth of a certain number of interdental plaque bacteria. The results also suggested a variable sensitivity of dental plaque bacterial communities to violet LED irradiation, and that the composition of oral microbiota may shift with respect to irradiation intensity. Furthermore, owing to the various bacterial compositions of individuals, the patterns of microbial responses appeared to differ among individuals [33]. Although the findings of this study could not completely reflect the oral microbial ecosystem, we still believe that these results have important implications for understanding shifts in dental plaque microbiota in response to violet LED irradiation.

Owing to the fact that antibiotic-resistant bacteria are increasing globally in recent years, researchers have proposed antimicrobial therapies based on PDT, which has a lower risk of emergence of these resistant bacteria [34]. PDT generates reactive oxygen species (ROS) that destroy the targeted bacteria, though the process requires the application of a photosensitizer dye molecule activated by a laser generating light at a specific wavelength under aerobic conditions [35]. Currently, PDT is being investigated for the treatment of infectious oral diseases [25,26,27]. In most clinical studies, red light (approximately 630 nm) is chosen as the excitation source to obtain a deeper penetration depth [36,37]. Conversely, we used violet light with a wavelength of approximately 405 nm, which is applied in resin restoration, tooth whitening, and other oral treatments [38,39], because of its shallower penetration depth; thus, this light wavelength may avoid damaging deep healthy tissues, especially when acting on microbiomes of superficial tissues such as periodontal pockets. Furthermore, PDT induced by violet LED irradiation at approximately 405 nm inactivates a range of medically important bacteria without requiring previous delivery of exogenous photoactivated compounds [30,31,32]. Here, for the first time, we explored structural shifts in the bacterial microbiota in response to violet light irradiation alone. Interdental plaque was chosen as the target for treatment with violet LED irradiation. The use of laser light to more efficiently disinfect the periodontal pocket has been an area of intense research [40,41]. Located between periodontal tissue and adjacent teeth, dental plaque at the interdental site is known to be obstinate to remove; therefore, these accumulations are prone to dental caries and periodontal diseases [42,43]. It is strongly advocated to maintain (or alter) the oral microbiome at (or to) a relatively healthy pattern by combining interdental plaque removal with existing toothbrushing habits; however, long-term compliance remains an issue [44]. The results of our experiments suggest that PDT induced by violet LED irradiation at approximately 405 nm is a useful accompaniment to these treatments for maintaining the stability of the microbiota around periodontal tissues. Nevertheless, this approach remains to be investigated.

Our previous study showed that the alpha diversity of plaque microbiota decreased with the addition of a nitric oxide donor in vitro [22]. After in vitro violet LED irradiation in this study, the alpha diversity of the plaque community also decreased significantly. One of the reasons for this change in the diversity of dental plaque microbiota may be the decrease in the proportion of *Fusobacterium* species in the community. This is consistent with the results of a previous experiment, in which multispecies biofilms were irradiated with blue light (142 J/cm^2^) [29]. In the current study, *F. periodonticum* and *F. nucleatum* were highly sensitive to violet LED irradiation, regardless of their subspecies classification. Their abundance in the microbiota was significantly reduced after in vitro irradiation in interdental plaque. Acting as a major bridging organism with coaggregation ability connecting early and late colonizers, *F. nucleatum* plays a key role in the formation and maturation of oral biofilms [45]. *F. nucleatum* is not only associated with periodontal disease, but is also suggested to have a potential role in colorectal cancer development and progression [45,46,47,48]. In addition, exposure to blue light with a wavelength of 400–500 nm may have a phototoxic effect on *F. nucleatum* in multispecies biofilms [29]. If the proportion of *F. nucleatum* in the oral cavity can be controlled via violet LED irradiation, it may potentially prevent the development of periodontal disease and colon cancer [49,50]. The detailed mechanism of *Fusobacterium* species reduction by violet LEDs could not be clarified in this study. However, in a previous study, the bactericidal effect of PDT using a light source similar to the wavelength used in this study was tested against Gram-negative anaerobic oral bacteria such as *P. gingivalis*, *Prevotella species*, and *F. nucleatum* subspecies [28]. The results identified protoporphyrin IX (PpIX) as the cause of this bactericidal effect [28]. As a precursor of heme, PpIX, though in small amounts, is ubiquitous in almost all living cells [51] and has a strong absorption band called the Soret band in the violet spectral region located around 405 nm, which is 10 times stronger than the absorption at 630 nm [52]. PpIX then absorbs light energy and transfers this energy to oxygen to generate singlet oxygen, which can cause cytotoxicity via oxidation of bacterial DNA [51]. Guanosines, as a constituent DNA base, were specifically hydroxylated at the C-8 position when exposed to ROS, generating 8-OHdG, which is commonly used as an indicator of oxidative DNA damage induced by ROS, due to its stability [53]. It has been reported that PpIX acting as an endogenous photosensitizer is activated by 460 nm light to generate 8-OHdG related to the production of ROS, which inhibits the bacterial growth of *P. gingivalis* without the addition of dyes for the phototoxic effect [54]. In this study, we evaluated oxidative stress after LED irradiation and found that 8-OHdG in DNA was significantly increased in *F. nucleatum* subsp. *polymorphum* irradiated at 50 J/cm^2^, compared to the nonirradiated group. These results and previous reports suggest that violet LED irradiation produces singlet oxygen from PpIX in *F. nucleatum*, which may cause oxidative damage to DNA and inhibit the growth of *F. nucleatum* [53,54]. We also found that oral *Veillonella* species were resistant to violet LED irradiation, and their abundance in the microbiota was increased by the violet LED irradiation of plaque and in vitro culture. The *Veillonella* spp. are Gram-negative cocci that require a strictly anaerobic environment, which is a common constitution of normal microbiota of the intestinal, respiratory, gastrointestinal, and oral tracts of humans [55]. Due to their ability of neutralizing acids, *Veillonella* species are considered as beneficial microorganisms for caries prevention [56]. Several reports have shown that most species of *Veillonella* possess a putative catalase gene that plays an important role in the defense against ROS through H_2_O_2_ removal [57,58], which may allow *Veillonella* to avoid the bactericidal effect of violet LED irradiation. In our previous study, we also reported that *C. concisus* showed high oxidative stress tolerance [22]. These differences in oxidative tolerance may be responsible for the difference in sensitivity to violet LEDs. Detailed analysis of the resistance mechanisms of individual bacteria will be important for the future clinical application of bacterial microbiota control.

Thus, PDT is an attractive alternative to antibiotic therapy for solving the problem of antibiotic resistance [59,60]. Recent studies by Tomb et al. have shown that even under repeated irradiation of 405 nm light, resistance of specific microorganisms is unlikely to occur [59]; as such, further applications of 405 nm light for the purpose of clinical treatment and decontamination are expected in the future [61,62]. However, as violet light itself brings oxidation stress to oral tissues, the aggressive administration of antioxidants prior to light treatment is advocated to prevent or mitigate the resulting damage to oral tissues [63]. Moreover, the samples used in this study were not dental plaques isolated from the foci of dental caries or periodontal disease. In the future, we will be able to elucidate the characteristics of the oral microbiota by observing the changes induced by violet LED irradiation in these samples that are in a state of dysbiosis. In addition, a recent review reported that irradiation with a wavelength between 400 and 470 nm may serve another useful purpose of alleviating opportunistic bacterial infections relevant to coronavirus infections, including coronavirus disease (COVID-19) [64]. Although violet light has been considered as a novel antimicrobial agent for directly killing bacteria, this study showed that violet light may also serve as a preventive measure to improve human health by maintaining and restoring a balanced human oral microbiome shift.

## 4. Materials and Methods

### 4.1. Sample Collection

The study participants included 12 adults (eight males, four females) within the ages of 24 and 45 years (mean ± SD, 27.8 ± 5.5). The characteristics of each subject are presented in Table S2. The Ethics Committee of Osaka Dental University reviewed and approved the protocol of this study (approval no.: 111002). All experiments and data collection were conducted according to the relevant guidelines and regulations. The informed consent form and acknowledgement of willingness to participation were acquired from all participants. Exclusion criteria included the following conditions: the self-reported presence of periodontal disease or dental caries, use of dental floss, interdental toothbrushes and/or other interdental care products within the three days prior to participation, use of mouth rinses within the week prior to participation, local or systemic treatment with antibiotics within the month prior to participation, a previous history of periodontal treatment within the past 6 months, and orthodontic treatment in progress (Table S3). All participants refrained from eating or drinking for 1 h before sample collection.

Dental plaque samples were collected from several interdental spaces using a disposable dental floss with a handle (Okina, Osaka, Japan). Plaques from each subject were extracted from dental floss by pipetting using 100 µL of phosphate-buffered saline (PBS, pH 7.4), and then transferred to a sterile plastic collection tube. Samples were cooled down by placing on ice immediately and repeating up-and-down pipetting with a narrow tip for homogenization under an anaerobic atmosphere of 80% nitrogen, 10% hydrogen, and 10% carbon dioxide. Cultivation was performed within 1 h of dental plaque collection.

### 4.2. Lighting Source and Conditions 

The excitation lighting source used in this study was an Aladuck^®^ LS-DLED (SBI Pharmaceuticals Co., Ltd., Tokyo, Japan), providing violet light through an LED with a peak at 400–410 nm. The LED output power was set to a constant intensity of 0.18 W/cm^2^ using a portable power/energy meter (Astral AI310, Scientech Inc., Boulder, CO, USA) before each experiment. Each well of a 12-well plate (Falcon^®^, Corning Inc., Corning, NY, USA) containing a bacterial suspension was irradiated with an LED from the top of the plate for up to 5 min.

### 4.3. Treatment by Irradiation and Culturing of Plaque-Derived Microbiota

After dental plaque collection, 100 µL of plaque suspensions was precultured overnight along with shaking under anaerobic conditions at 37 °C in 500 µL of SHI medium, which specializes in sustaining a microbial community that highly resembles the profile of the original oral microbiota [65,66,67,68]. After centrifugation and removal of the supernatant, the resulting pellet was resuspended in 500 µL of PBS. Aliquots (300 µL) of suspensions were collected as initial samples and then stored at −80 °C, while 50 µL of suspensions was adjusted with PBS to an optical density at 600 nm (OD 600) of 0.1. The suspensions were then inserted into a 12-well plate at the amount of 1 mL per well and irradiated at 0, 10, 25, or 50 J/cm^2^, corresponding to exposure times of 0, 56, 139, or 278 s, respectively. After irradiation, 100 µL of samples was suspended in 900 µL of SHI medium and cultivated for 20 h at 37 °C along with shaking under anaerobic conditions. After centrifugation on all cultures, only pellets were saved as samples and stored at −80 °C. DNA extraction was then performed within one week.

### 4.4. DNA Extraction 

Metagenomic DNA extraction was then performed on the frozen pellets via chemical and mechanical lysis using a Pathogen Lysis Tube S and QIAamp UCP Pathogen Mini Kit (Qiagen, Hilden, Germany) [22,23,69]. Subsequently, quantification of the purified genomic DNA was performed using a Quantus fluorometer (Promega, Madison, WI, USA) and Qubit dsDNA BR Assay Kit (Thermo Fisher Scientific, Waltham, MA, USA). The quantified DNA was frozen at −80 °C until use.

### 4.5. Library Construction and High-Throughput Sequencing

Next-generation sequencing library preparation and sequencing were performed according to the protocol of the 16S metagenomic sequencing library preparation (Part # 15044223 Rev. B) from Illumina (San Diego, CA, USA). Briefly, DNA amplification targeting the V3–V4 region of the 16S ribosomal RNA (rRNA) gene was conducted using PCR with primers 341F (5′-TCGTCGGCAGCGTCAGATGTGTATAAGAGACAGCCTACGGGNGGCWGCAG-3′) and 806R (5′-GTCTCGTGGGCTCGGAGATGTGTATAAGAGACAGGGACTACHVGGGTWTCTAAT-3′) (custom-synthesized by Invitrogen), along with Premix Ex Taq polymerase (Takara Bio, Otsu, Japan). The thermal cycling protocol was as follows: initial denaturation for 10 s at 98 °C, followed by a circulation of 10 s at 98 °C, 30 s at 55 °C, and 1 min at 72 °C, running 25 times. Electrophoresis on a 1% agarose gel was used to check the DNA integrity. After PCR amplification, replicate amplicons were purified via AMPure XP beads (Beckman Coulter, Miami, FL, USA). Additional PCR amplification was performed for eight cycles, as with the conditions described above, to ligate the purified DNA amplicons on both 3′ and 5′ ends with the adaptor primer containing 8 bp indices. The PCR product was then purified with AMPure XP beads, followed by quantification using a Quantus fluorometer and a Qubit dsDNA HS Assay Kit (Thermo Fisher Scientific, MA, USA). To create the final library, the amplicons were pooled in equimolar amounts and mixed with 5% equimolar amounts of PhiX DNA (Illumina, USA). After library construction, 16S rRNA gene sequencing was performed at 2 × 250 bp paired-end reads using the Illumina MiSeq platform (Illumina, USA) at the Oral Microbiome Center in Takamatsu, Japan.

### 4.6. Sequence Data Processing

Demultiplexed paired-end reads from the sequencing step above were processed using QIIME 2 (the Quantitative Insights Into Microbial Ecology 2) version 2021.2 pipeline [70]. Metadata files were verified for formatting using Keemei [71]. Filtering for quality and trimming were performed on the raw reads using DADA2, along with chimeras filtered by quality and consensus (via q2-dada2) [72] using the following parameters: trim-left-f = 17; trim-left-r = 21; trunc-len-f and trunc-len-r were installed as 252 and 254, respectively, according to the sequence quality. The resulting exact ASVs were then merged into one single feature table with the usage of the q2-feature-table plugin. A naïve Bayes taxonomy classifier (via q2-feature-classifier) [73] was trained, based on the V3–V4 region of the 16S rRNA sequences in the expanded human oral microbiome database (eHOMD; v.15.2) [74] to apply to each ASV for taxonomic assignment. With the usage of MAFFT [75] (via q2-alignment), all ASVs were aligned and were then used for constructing a phylogeny with FastTree 2 (via q2-phylogeny) [76]. Visualization of genus abundances and correlations were plotted by combining the functions provided by the ampvis2 (v.2.7.5) [77] and ggplot2 [78] R packages. Analyses were conducted using R (v.4.0.1) and RStudio (v.1.3.959).

### 4.7. Absolute Quantification by Quantitative PCR Analysis

To assess the total number of bacterial DNA, quantitative PCR was performed using the universal bacterial 16S rRNA primers, i.e., 339F (5′-ACTCCTACGGGAGGCAGCAGT-3′) and 514R (5′-ATTACCGCGGCTGCTGGC-3′). 16S RNA genes were extracted from *Actinomyces oris* MG1 and were then ligated to vector plasmid pMD20-T using the Mighty TA-cloning Kit (Takara Bio, Shiga, Japan), to establish a real-time PCR control. The MiniOpticon platform was used for amplification with the QuantiFast SYBR Green PCR Master Mix (Qiagen GmbH, Hilden, Germany). Latter analyses were performed by Bio-Rad CFX Manager 1.5.

### 4.8. Bacterial Strains and Culture Conditions

*V. parvula* JCM 12972 and *F. nucleatum* subspecies *polymorphum* JCM 12990 were cultured anaerobically under 80% nitrogen, 10% hydrogen, and 10% carbon dioxide at 37 °C, using modified GAM (Gifu anaerobic medium; Nissui Pharmaceutical, Tokyo, Japan).

### 4.9. Bactericidal Viability Assay

*F. nucleatum* strains were precultivated under anaerobic conditions in 6 mL of modified GAM medium for 20 h at 37 °C. Cultured samples were washed and resuspended with PBS buffer, and then adjusted with the same buffer to OD 600 of 0.1. The suspensions were then inserted into a 12-well plate at the amount of 1 mL per well and irradiated at 0, 10, 25, or 50 J/cm^2^. Immediately after irradiation, serial dilutions were conducted on the bacterial suspension. An Eddy Jet2 (IUL Instruments, Barcelona, Spain), a spiral plater, was used to apply the diluted samples on a modified GAM plate. The inoculated plates underwent incubation under anaerobic conditions at 37 °C for 3–4 days, followed by the operation of colony counts, which was performed with an automated plate counter (aCOLyte3, Synoptics, Cambridge, UK). The same test was performed when studying *V. parvula*, but with the usage of the spot-plating technique [79] instead of the spiral plater.

### 4.10. Analysis of DNA Oxidation by ELISA

After anaerobic precultivation in 6 mL of modified GAM medium at 37 °C for 20 h, the suspension of *F. nucleatum* cells was adjusted to OD 600 of 0.5, and aliquots of 1 mL were placed in a 12-well culture plate. The samples were then irradiated with a violet LED at 0 or 50 J/cm^2^. DNA extraction of the precipitates, obtained by centrifugation from the samples, was performed using the NucleoSpin Microbial DNA kit (Macherey-Nagel, Düren, Germany). The total extracted DNA from each specimen was quantified and qualified using a NanoDrop ND-1000 (NanoDrop Technologies, Thermo Scientific, Wilmington, DE, USA). Nuclease P1 was used to digest the extracted DNA, with the use of the 8-OHdG Assay Preparation Reagent Set (Wako Pure Chemical Industries, Ltd., Osaka, Japan). 8-OHdG levels were measured using a competitive ELISA kit (Highly Sensitive 8-OHdG Check, Japan Institute for the Control of Aging, Shizuoka, Japan), following the manufacturer’s instructions. The absorbance value was measured on an ELISA plate reader set at 450 nm with a SpectraMax M5 plate reader (Molecular Devices, CA, USA). Then, compared to a standard curve, the concentrations of 8-OHdG in all samples were calculated.

### 4.11. Statistical Analyses

Statistical significance in quantitative PCR was analyzed using the Wilcoxon rank-sum test. Alpha (intrasample) diversity was calculated using the QIIME 2 q2-diversity plugin with the Shannon index, observed ASVs, and Pielou’s evenness index. Comparisons across experimental groups were performed by conducting a Kruskal–Wallis test. Beta (intercommunity) diversity calculations were conducted using the weighted UniFrac phylogenetic distance (via q2-diversity), which was then visualized using EMPeror (via q2-emperor) as two-dimensional principal coordinate analysis plots. Significant differences in bacterial composition among samples were tested using permutational analysis of variance. The linear discriminant analysis effect size [80] was used to evaluate the differentially abundant bacterial taxa. The OTU table (Table S4) was analyzed by the Rhea pipeline, which provides numerous downstream analysis choices encoded in a set of R scripts. OTUs with significant abundance changes were then extracted using the same pipeline [81]. The calculation of *p*-values in differential abundance analysis at the OTU level was performed using the Wilcoxon rank-sum test and Fisher’s exact test. In the bacterial viability assay and oxidative stress analysis, *p*-values were calculated using Student’s *t*-test. A *p*-value less than 0.05 was considered significant for all analyses. *p*-values less than 0.05 are shown in this paper.

### 4.12. Availability of Data

All raw sequence data generated during this research are accessible from the DNA Data Bank of Japan under the accession number DRA012915 (http://www.ddbj.nig.ac.jp/, https://www.ebi.ac.uk/ena/browser/view/, accessed on 4 November 2021).

## 5. Conclusions

To the best of authors’ knowledge, this was the first study to reveal that the structure of the oral microbial community shifts under violet LED irradiation through a combination of in vitro cultivation techniques and high-throughput sequencing. This research also identified several bacterial OTUs responding to violet LED irradiation according to each subject’s ecosystem. According to our results, 405 nm violet light technology represents a novel approach for maintaining a balanced oral microbiome shift and may even contribute to overall human health. However, the sample size of this study was small and the environment of sampling and irradiation was different from the original oral cavity. In the future, further understanding the bacteria–bacteria interaction in the changed oral microbiome in different doses may give more insight into the microbial associations in the oral cavity.

## Data Availability

All raw sequence data generated during this research are accessible from the DNA Data Bank of Japan under the accession number DRA012915 (http://www.ddbj.nig.ac.jp/, https://www.ebi.ac.uk/ena/browser/view/, accessed on 4 November 2021).

## Supplementary Materials

The following are available online at https://www.mdpi.com/article/10.3390/antibiotics10111348/s1, Figure S1: Rarefaction curves, Figure S2: Stacked bar charts, Figure S3: Unifrac distances, Table S1: *p*-value list, Table S2: Characteristics of the subjects, Table S3: Inclusion and exclusion criteria, Table S4: OTU table.
