# Supplementary material for: Interdental Plaque Microbial Community Changes under In Vitro Violet LED Irradiation
Source: Antibiotics (Basel). 2021 Nov 4;10(11):1348. doi: 10.3390/antibiotics10111348 (PMC8614803; doi:10.3390/antibiotics10111348)
Supplement: Supplementary file 1 [file antibiotics-10-01348-s001.zip › SM/supplementary figures.pdf]

Supplementary

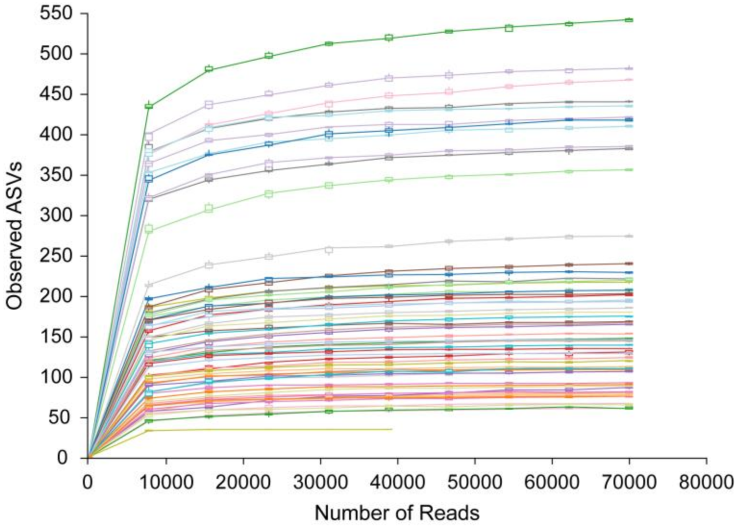

Figure S1: Rarefaction curves based on alpha-diversity metrics.

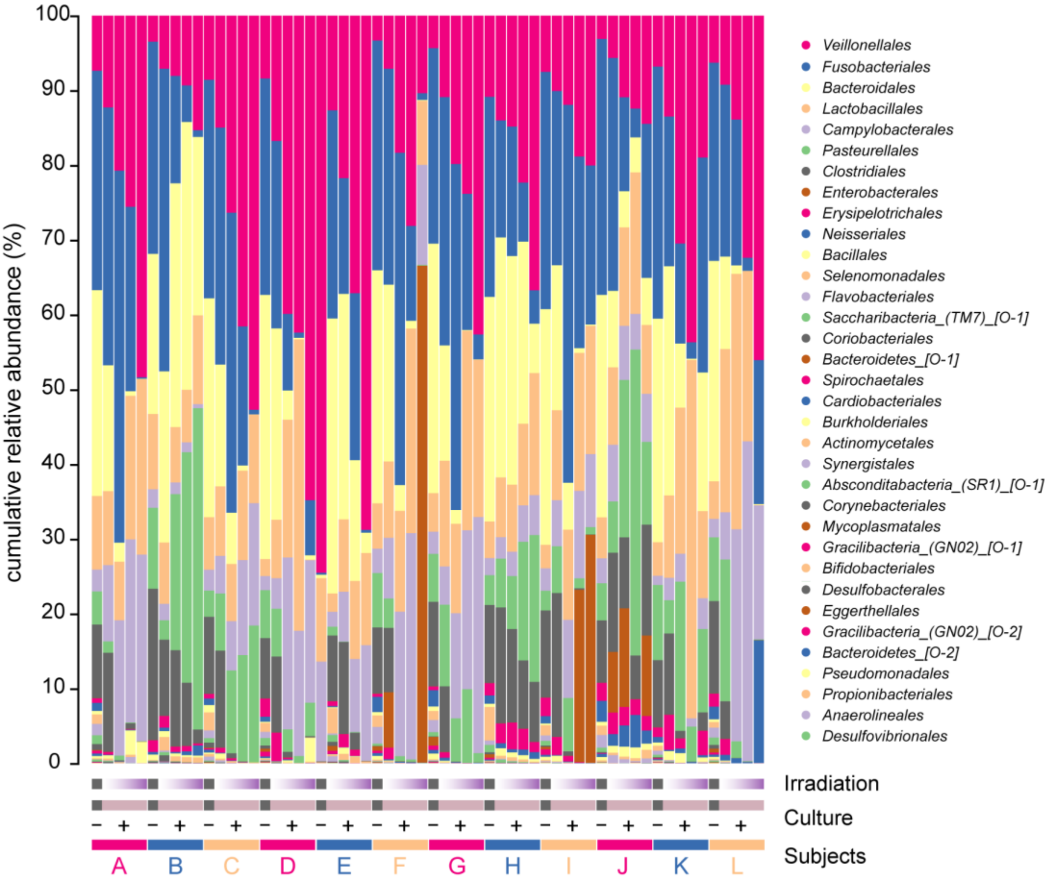

Figure S2: Stacked bar charts visualizing the changes in the bacterial composition of plaque microbiota at the order level under violet LED irradiation and in vitro culture. Each bar in the chart represents a single sample, and segments in the bar represent different relative abundance of different bacterial taxa. Data are sorted by individual (A~L).

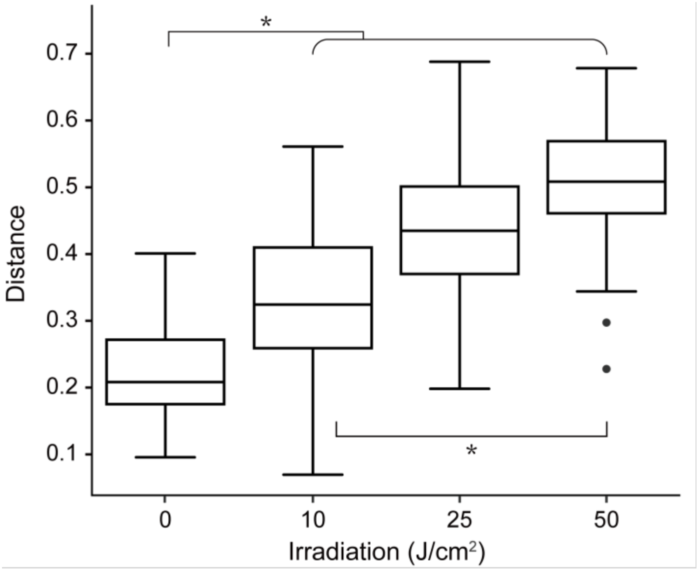

Figure S3: Unifrac distances from the 0 J/cm² irradiated sample to each sample (* $p < 0.01$, pairwise permutational analysis of variance).
